# Mapping of Lapping-Induced Subsurface Damage in Planar Fused Silica Glass Based on Polarized Laser Scattering Method

**DOI:** 10.3390/ma18112417

**Published:** 2025-05-22

**Authors:** Mingchuan Gao, Yi Guo, Chenxi Liu, Chuanxin He, Qian Bai

**Affiliations:** State Key Laboratory of High-Performance Precision Manufacturing, Dalian University of Technology, Dalian 116024, China; 2060934279@mail.dlut.edu.cn (M.G.); 3413520386@mail.dlut.edu.cn (Y.G.); 723918643@mail.dlut.edu.cn (C.L.); 1416122356@mail.dlut.edu.cn (C.H.)

**Keywords:** fused silica glass, subsurface damage, polarization laser scattering detection, mapping

## Abstract

Fused silica glass is a critical material in many industries due to its superior physicochemical properties. The detection of subsurface damage (SSD) poses fundamental challenges that directly affect the performance of fused silica glass. The polarized laser scattering (PLS) detection method has significant advantages in SSD detection, but damage mapping has not yet been achieved. This paper proposes an SSD mapping method based on the PLS detection results. The relationship between the PLS detection signals and the SSD depths of fused silica glass is established, and an SSD mapping diagram is successfully generated. Unlike existing studies that only provide local quantitative SSD depth, SSD mapping achieves simultaneous visualization of SSD location, depth, and uniformity of SSD distribution across the entire region, which provides guidance to determine the lapping or polishing parameters in the subsequent processes.

## 1. Introduction

Fused silica glass, composed of Si-O bonds, is a non-crystalline solid with short-range order and long-range disorder [1]. This structure endows it with exceptional physicochemical properties, such as excellent chemical stability [2,3], high strength [4], and superior high-temperature resistance [5,6,7], which make fused silica glass a crucial material for aeronautics [8], high-end optical systems [9], and other fields.

Lapping is a key technology in producing fused silica glass parts [10]; however, due to the hard and brittle characteristics of fused silica glass, it is difficult to avoid the generation of subsurface damage (SSD) during processing [11]. SSD significantly affects the stability and optical properties of fused silica glass. SSD detection is crucial for optimizing the machining parameters to achieve high-efficiency and low-damage parts [12]. Thus, research on the SSD detection of fused silica glass is essential for advancing its application in high-tech fields.

Existing methods for SSD detection in hard and brittle materials include both destructive and non-destructive methods [13]. Destructive methods such as etching [14,15], taper polishing [16,17,18], magnetorheological finishing (MRF) [19], cross-sectional microscopy [20,21], and transmission electron microscopy (TEM) [22,23] have a critical limitation in that they introduce new damage to the components during chemical etching, polishing, or cutting, which is unacceptable for subsequent use and performance evaluation. Traditional non-destructive detection methods include scanning acoustic microscopy [24], laser-ultrasonic technology [25,26], fluorescence confocal [27], and photothermal weak absorption [28,29]. Scanning acoustic microscopy has a high detection efficiency, but it requires high surface quality. Liu et al. [30]. utilized laser-ultrasonic technology to achieve the detection of SSD in ground silicon wafer, and the experimental results showed that when applying this method, the ratio of the material’s surface roughness to the minimum wavelength of laser-excited surface acoustic waves needs to be very small, approximately less than 0.01. The fluorescence confocal method enables global scanning; however, enhancing its longitudinal and transverse resolution necessitates a trade-off with reduced signal intensity, which ultimately limits its effectiveness in detecting SSD [31]. Photothermal weak absorption has high sensitivity, but its low resolution prevents the detection of micron-level SSD [32]. Polarized laser scattering (PLS) detection leverages a change in the polarization state of polarized light to distinguish SSD from surface roughness (Ra), offering a non-destructive approach immune to the Ra effect [33]. Additionally, its high lateral and longitudinal resolution enables the effective detection of deeper SSD, making it particularly suitable for SSD detection in high-precision components. Yin et al. [34] used this method to achieve the SSD detection of silicon wafers lapped with #20000 grit wheels. Ma et al. [35] applied this method to perform a two-dimensional scan in the depth direction on fused silica glass samples with SSD depths ranging from 20 to 35 μm, and the error was within 5%. However, the SSD mapping of lapped fused silica glass has not been realized. This study builds a detection platform based on the PLS detection method, and proposes a method to achieve the SSD mapping, which provides theoretical guidance for lapped fused silica glass SSD detection.

## 2. Lapping Experiment Materials and Process

**Figure 1:**
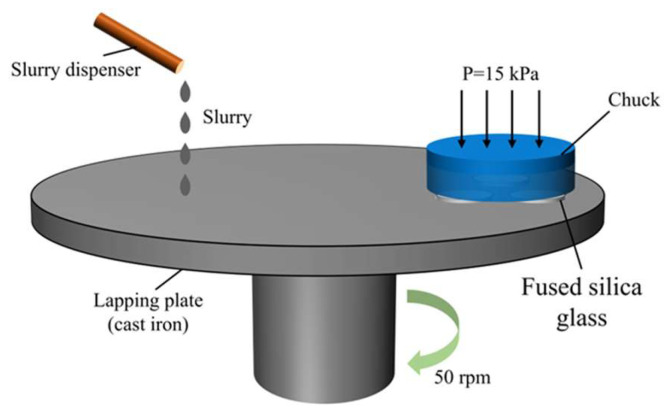
Schematic diagram of lapping.

A lapping experiment was conducted to generate different depths of SSD for fused silica glass. The materials used in the lapping experiment were double-side polished Corning glass wafers with a diameter of 25 mm and a thickness of 0.8 mm. A lapping machine (HD-380X, Hyder, Shenzhen, China) with a self-trimming function was used for the lapping process. A chuck with a weight of 1 kg applied pressure to the fused silica glass samples, with the chuck itself having a diameter of 130 mm and a thickness of 22 mm. The chuck surface was machined with three grooves of 30.5 mm in diameter and 0.2 mm in depth, and the fused silica glasses were fixed in the grooves of the chuck by wax. Before lapping, the self-trimming was used to ensure the surface precision of the lapping plate. The schematic diagram of lapping is shown in Figure 1.

In this study, fused silica glasses were lapped using the loose abrasive lapping method, and diamond abrasive lapping slurry (SO1-1104D, Zhongwei, Nanning, China) was selected. Grit size, lapping pressure, and plate rotation speed were identified as key factors notably affecting the SSD depth of fused silica glass [36]. Among these factors, grit size exhibited the most pronounced effect [20]. In this experiment, the grit sizes of 5 μm, 10 μm, 15 μm, and 20 μm were used to cover typical industrial scenarios where different grits balance efficiency and surface quality [37], with larger grits (15 μm and 20 μm) simulating high-efficiency rough grinding and smaller ones (5 μm and 10 μm) mimicking precision grinding [32]. An additional group of as-received fused silica glass, i.e., double-side polished fused silica glass, was also detected for the signal comparison. The experiments were conducted in five groups, with the parameters in Table 1 kept unchanged. All parameter values in Table 1 were chosen to obtain uniform surface roughness (Ra).

**Figure 2:**
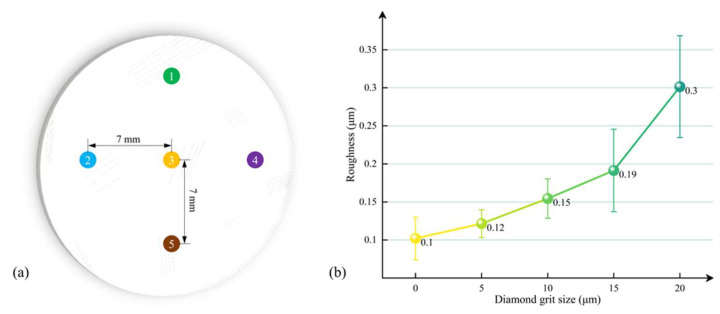
(**a**) Locations of measured points (1–5) for Ra; (**b**) Ra values corresponding to different diamond grit sizes.

A white light interferometer (New-View 9000, ZYGO, Fremont, CA, USA) was used to measure the Ra of fused silica glass, with each sample being measured at five designated positions as illustrated in Figure 2a. For each sample, the average Ra of these five positions was taken as shown in Figure 2b. The Ra of the as-received fused silica glass is 0.1 μm. When the diamond grit size increases to 20 μm, the Ra of the lapped fused silica glass increases to 0.3 μm. The Ra increases with the diamond grit size, exhibiting a positive correlation between the two. This correlation is consistent with the experimental results of Lv et al. [38].

## 3. Detection Platform for SSD of Fused Silica Glass

### 3.1. PLS Detection Method

**Figure 3:**
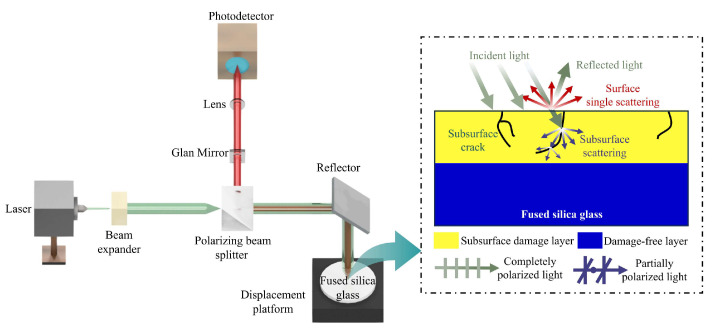
Schematic of PLS detection method.

The principle of the PLS detection method is illustrated in Figure 3. Contrary to silicon wafers, the stress birefringence effect of fused silica glass is extremely low [39]. Therefore, a fixed P-polarization laser beam was used without considering stress orientation effects. The P-polarized light emitted by the laser is incident at a certain angle onto the fused silica glass surface. At the roughness and crack sites on the surface, single scattering occurs with polarization preservation, meaning the polarization state remains unchanged. However, when the P-polarized light reaches the SSD, the light undergoes depolarization due to the influence of the cracks. The deeper the crack, the stronger the depolarization. The light returning along the original path contains both P-polarized light and partially polarized light, as well as S-polarized light. The S-polarized light carries SSD information. Detecting this light using a photodetector enables the SSD analysis.

### 3.2. PLS Detection System

**Figure 4:**
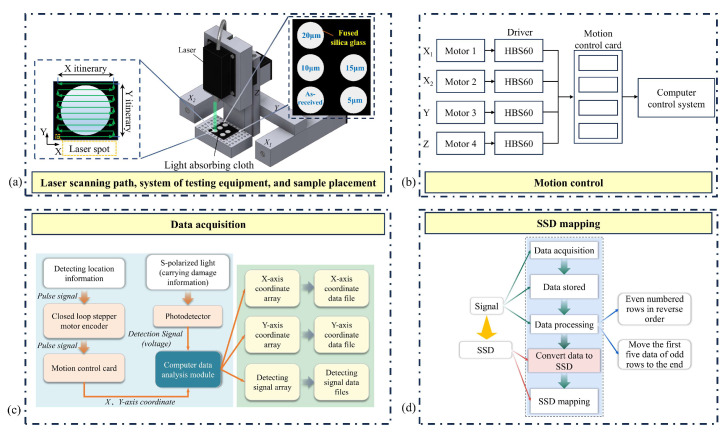
PLS detection system: (**a**) Laser scanning path, system of testing equipment, and sample placement; (**b**) Motion control subsystem; (**c**) Data acquisition subsystem; (**d**) SSD mapping subsystem.

Consisting of four motion axes and an optical system mounted on the Z-axis, the PLS detection system is shown in Figure 4a. It has been found that a shorter laser wavelength helps to improve the scattering effect [40], but if the wavelength is too short, the laser penetration will be weakened, making it difficult to detect deep SSD and thus reducing the detection accuracy. To ensure sufficient scattering detection efficiency and good light penetration, this study selected a laser (MDL-III-405, CNI Laser, Changchun, China) with a wavelength of 405 nm and with a laser spot diameter of 2 mm. At the same time, the laser power can be adjusted in the range of 1–150 mW, and the power stability less than 3%, to meet the PLS detection process requirements. A polarized beam splitter (PBS25-532, Thorlabs, Newton, NJ, USA) with a high extinction ratio (transmittance of P-polarized light (Tp)/transmittance of S-polarized light (Ts) > 2000:1) was used. The photodetector (S121C, Thorlabs, Newton, NJ, USA) converted the optical signal into an electrical signal. The Glan mirror (PGT5101, Dayoptics, Fuzhou, China) separated the S-polarized light at 405 nm, allowing the photodetector to detect the S-polarized light that carried information about the SSD.

During the detection process, the test sample remained stationary while the detection system moved along an S-shaped trajectory, as shown in Figure 4a, which enables the continuous detection and reduced the problem of mechanical drift. In this study, the Y-axis stroke was 90 mm and the diameter of the laser spot was 2 mm. In order to realize the full-area detection of the parts, the movement increment along the X-axis was set to 1 mm.

The motion control of the detection system was realized using a closed-loop stepper motor (57 × 112, Moons’, Shanghai, China), motor driver (HBS60, Haijie, Beijing, China), and motion control card (PCIE_AMC2XE, Hengkai, Puyang, China), as shown in Figure 4b. The driver was used to control the rotation of the stepper motor. Since the closed-loop stepper motor cannot directly receive digital signals, the driver controlled the rotation angle and speed of the motor based on the pulse signals it received. The motion control card received commands from the computer system, converted them into driver-required pulse signals, and transmitted these signals to the driver to control the movement of the motor. Once the computer received the operator’s start command, the system program continuously sent motion commands to the motion control card to drive fixed-stroke movements of the axes. This enabled the PLS detection platform to follow a predefined trajectory within a 90 mm × 60 mm range.

The data acquisition of the system consisted of two components: position data and detection signal, as shown in Figure 4c. The position data was fed back to the motion control card via the encoder of the closed-loop stepper motor in the form of pulse signals. The card then utilized its own 32-bit counter to convert these pulses into digital signals, which were transmitted to the data analysis module of the computer. Detection signal data was generated by the photodetector from Thorlabs, which converted the S-polarized light signals into electrical signals and sent them to the data analysis module. The data analysis module converted the collected coordinates and detection signals into easily stored and readable arrays.

Figure 4d illustrates the SSD mapping flowchart. By using the relationship between the SSD depth and the detection signal established in Section 4.1, the detection signals were converted into the SSD depths, thereby obtaining the SSD mapping diagram.

## 4. Experimental Results and Discussion

### 4.1. Analysis of Detection Results

**Figure 5:**
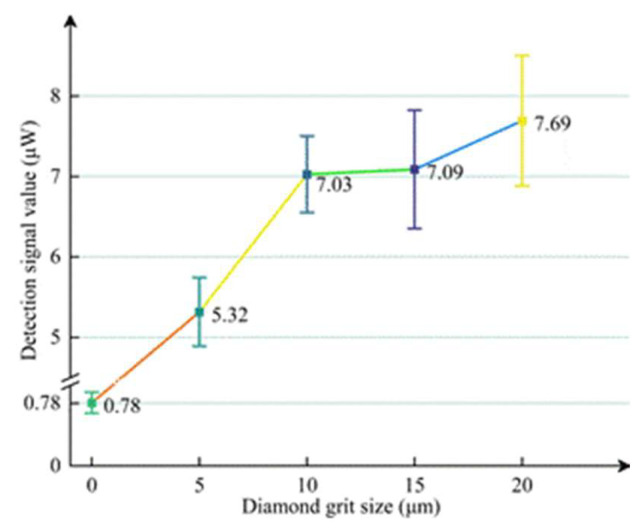
Detection signals corresponding to different diamond grit sizes.

Figure 5 presents the experimental results from the PLS detection system applied to five samples. The control group exhibits a detection signal of 0.78 μW, indicating that its subsurface is essentially undamaged. When the grit size increases to 20 μm, the signal increases to 7.69 μW. Data analysis reveals that as the grit size increases, the value of Ra for the fused silica glass also increases, leading to the increase of the PLS detection signal.

The value of Ra has strong correlation with SSD depth [41], which can be expressed as follows:

Depth of SSD = 9.1 Ra,(1)

**Figure 6:**
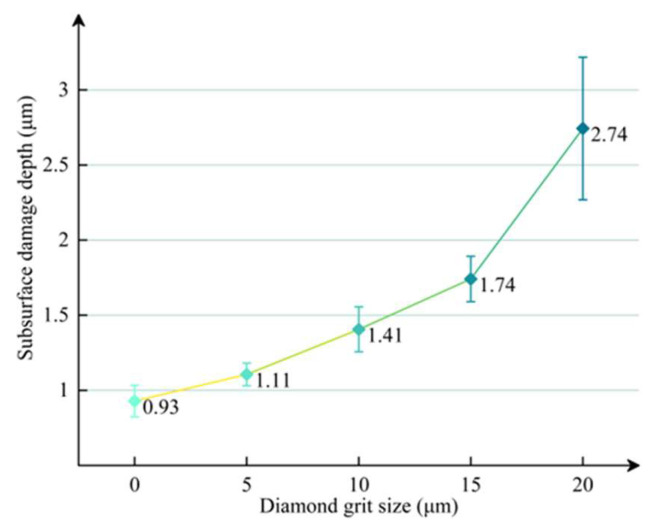
SSD depths corresponding to different diamond grit sizes.

Therefore, the SSD depths were calculated as shown in Figure 6.

**Figure 7:**
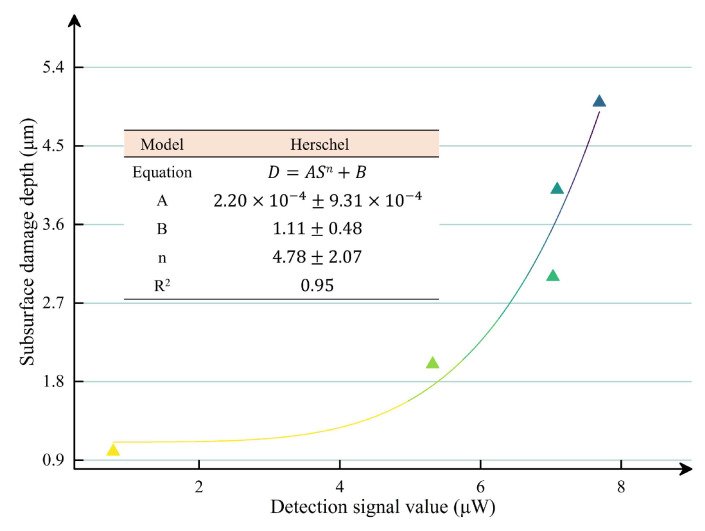
Relationship between SSD depth and detection signal.

The relationship between the SSD depth and the detection signal was established as shown in Figure 7. We selected the values of A, B, and n within the specified range to ensure that the errors between the calculated SSD and the experimental SSD were less than 10%, which can be expressed by Equation (2), with a correlation coefficient (R^2^) of 0.95.

D = 5.78 × 10^−6^ S^6.16^ + 0.86, (2)

where D represents the SSD depth and S represents the detection signal.

### 4.2. Validation and Error Analysis

**Figure 8:**
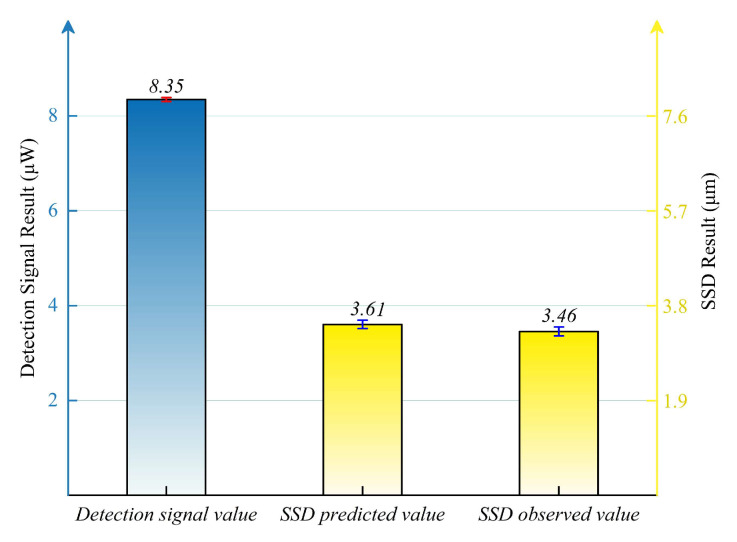
Results of validation experiments.

To validate the reliability of the established relationship between the PLS detection signals and the SSD depth, a validation experiment was conducted using a diamond grit size of 30 μm. The established system was used to measure the lapped sample, and the average of 3000 PLS detection signals obtained in one experiment yielded 8.35 μW, as shown in Figure 8. Using the fitting result in Section 4.1 to convert each detection signal to SSD depth yielded an average SSD depth of 3.61 μm. After calculating the standard error, we obtained the error bar, as shown in Figure 8. Meanwhile, the surface roughness (Ra value) of the sample was measured as 0.380 μm by the white light interferometer, and the SSD depth was alternatively calculated using Equation (1), resulting in 3.46 μm. The relative error between the two results was determined to be 4.3%. The small SSD error bar indicates that the uncertainty range of the SSD depth data converted from the PLS detection signals is small. Together with the small relative error, this supports the reliability of the relationship between PLS detection signals and SSD depths.

During the testing process, the power of the laser was unstable, which led to instability in the wavelength of the laser produced by the laser, thus leading to experimental errors. Therefore, a more stable power laser could be employed to improve the performance of the system in the future.

### 4.3. Fused Silica Glass SSD Mapping

The built platform was used to obtain the SSD mapping diagram of the five samples. The placement of the five samples is shown in Figure 4a. The moving speed of the detection probe is 5 mm/s, the data acquisition frequency is 20 Hz, and the number of acquisition cycles is 90.

**Figure 9:**
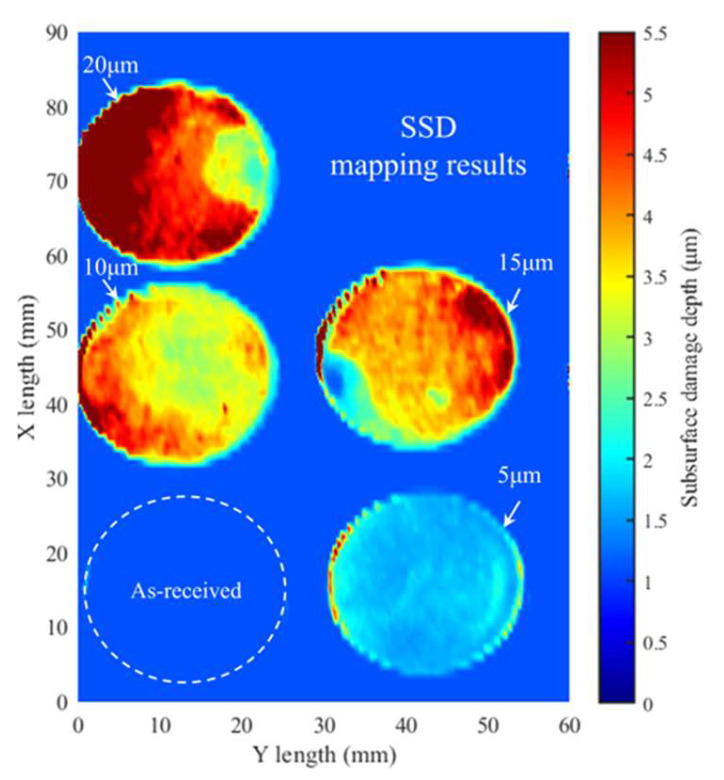
SSD mapping diagram.

Figure 9 shows the SSD mapping results. Figure 9 indicates that when the grit size is 5 μm, there is basically no damage. Then, with the increase of the grit size, the SSD degree deepens. When the grit size reaches 20 μm, the SSD degree reaches its maximum. The SSD distribution is uneven and there is a chipping phenomenon, which may be due to the uneven lapping pressure.

From Figure 9, the SSD mapping diagram enables the assessment of SSD severity through full-area visualization, thereby facilitating the classification of components into quality tiers. Based on this assessment, exact locations requiring SSD removal can be determined in accordance with product quality standards, optimizing material removal processes and reducing post-processing time. Additionally, the SSD mapping diagram allows for evaluating lapping quality by analyzing SSD distribution. For instance, the coexistence of severe and mild SSD in the same component may reveal process issues such as uneven lapping pressure, inappropriate lapping duration, or non-uniform lapping plate surfaces, enabling manufacturers to identify and address process inconsistencies to ensure uniform material removal and compliance with industry standards for component surface integrity.

In the future, the development of inline SSD detection devices during machining is essential. By integrating real-time SSD detection directly into machining workflows, it can minimize processing errors caused by repeated workpiece repositioning and eliminate time-consuming offline inspections, thereby enhancing both inspection efficiency and machining precision in real-time industrial processes.

## 5. Conclusions

In this paper, a PLS detection system including motion control, data acquisition, and SSD mapping was developed. The conclusions obtained are as follows:

1. Because deeper SSD enhances the depolarization of incident P-polarized light, generating stronger S-polarized signals that carry SSD information means that the PLS detection signal increases with increasing SSD depth, indicating a direct correlation between the PLS signal and SSD depth;
2. As the grit size increases to 20 μm, the SSD depth increases to approximately 2.74 μm. The relationship between the PLS detection signal and the SSD depth obtained through experiments was expressed as D = 5.78 × 10^−6^ S^6.16^ + 0.86, with an R^2^ value of 0.95;
3. Established system and fitting results can effectively detect SSD in fused silica glass with an error within 5%, and the SSD mapping diagram established based on the fitting result clearly visualized the SSD location, SSD depth, and lapping quality (e.g., lapping uniformity and edge integrity);
4. Future research will focus on realizing inline SSD detection during the processing of fused silica glass.

## Figures and Tables

**Table 1:** Partial lapping parameter.

Lapping Pressure	Plate Rotation Speed	Flow Rate of Slurry	Lapping Time
15 kPa	50 rpm	25 mL/min	50 min

## Data Availability

Data available on request due to restrictions. The data presented in this study are available on request from the corresponding author.
